# Novel Collaborative Weighted Non-negative Matrix Factorization Improves Prediction of Disease-Associated Human Microbes

**DOI:** 10.3389/fmicb.2022.834982

**Published:** 2022-03-10

**Authors:** Da Xu, Hanxiao Xu, Yusen Zhang, Rui Gao

**Affiliations:** ^1^School of Mathematics and Statistics, Shandong University, Weihai, China; ^2^School of Control Science and Engineering, Shandong University, Jinan, China

**Keywords:** microbe, disease, association prediction, collaborative weighted non-negative matrix factorization, graph Laplacian regularized least squares

## Abstract

Extensive clinical and biomedical studies have shown that microbiome plays a prominent role in human health. Identifying potential microbe–disease associations (MDAs) can help reveal the pathological mechanism of human diseases and be useful for the prevention, diagnosis, and treatment of human diseases. Therefore, it is necessary to develop effective computational models and reduce the cost and time of biological experiments. Here, we developed a novel machine learning-based joint framework called CWNMF-GLapRLS for human MDA prediction using the proposed collaborative weighted non-negative matrix factorization (CWNMF) technique and graph Laplacian regularized least squares. Especially, to fuse more similarity information, we calculated the functional similarity of microbes. To deal with missing values and effectively overcome the data sparsity problem, we proposed a collaborative weighted NMF technique to reconstruct the original association matrix. In addition, we developed a graph Laplacian regularized least-squares method for prediction. The experimental results of fivefold and leave-one-out cross-validation demonstrated that our method achieved the best performance by comparing it with 5 state-of-the-art methods on the benchmark dataset. Case studies further showed that the proposed method is an effective tool to predict potential MDAs and can provide more help for biomedical researchers.

## Introduction

Extensive clinical and biomedical studies have shown that microbiome has a prominent role in human health and disease. More than 100 trillion (10^14^) microbes inhabit the human gut and constitute a nutrient-rich environment where symbiotic relationships are of benefit to the host (Ley et al., 2006; Lozupone et al., 2012). Therefore, gut flora is often referred to as the “forgotten organ” (O’Hara and Shanahan, 2006). Once the balance is broken or the symbiotic relationship is disturbed, this close relationship will carry risks for the development of the disease, including cardiovascular disease (Wang et al., 2011), neurological disease (Tremlett et al., 2017), cancer (Schwabe and Jobin, 2013), inflammatory bowel disease (IBD) (Hossen et al., 2020), and so on. To better understand the medical and biological significance of the human microbiome, some large projects have been launched and made substantial progress, such as the project of metagenomics of the human intestinal tract (Ehrlich, 2011; Cho and Blaser, 2012) and the Human Microbiome Project (HMP) (Turnbaugh et al., 2007).

Studies investigating microbiomes demonstrated a critical role for microbes in the disease and health of humans. Considering the complexity and diversity of the microbial community, it is still a challenge to fully understand the interaction mechanism between microorganisms and human diseases, healthy composition, and functional states of the human microbiome. Because of the known disease-related microbes being insufficient, developing effective computational methods is necessary for reducing the cost and time of biological experiments. Recently, with the deepening of studies on computational biology, many computation-based methods have been proposed and achieved successful applications in the bioinformatics field, such as miRNA–disease (Peng et al., 2018a; Chen et al., 2019) or drug–target (Chen et al., 2016) association prediction, and lncRNA–miRNA (Zhang et al., 2021), protein–protein (Xu et al., 2020a), or lncRNA–protein (Peng et al., 2021; Zhou et al., 2021) interaction prediction.

Fortunately, in 2016, a human microbe–disease association database was constructed by Ma et al. (2017). It provided a foundation for identifying potential MDAs through computational methods. A basic assumption is mainly used in the developed methods that microbes will share similar interaction patterns with phenotype diseases if they have similar functions (Zhao et al., 2020). Chen et al. (2017) proposed the first computational model called KATZHMAD for MDA prediction using the KATZ measure. With the rapid development of artificial intelligence and machine learning (Camacho et al., 2018; Xu et al., 2020b), some machine learning-based models were proposed. For instance, Wang et al. (2017) developed the LRLSHMDA method using the Laplacian regularized least squares. In 2021, Xu et al. (2021b) developed a novel prediction model named MDAKRLS using multisimilarity and Kronecker regularized least squares for prediction and achieved better performance. Shi et al. (2018) designed a prediction model by binary matrix completion.

In addition, there are some network-based computational methods. For example, Zou et al. (2017) and Luo and Long (2020) developed BiRWHMDA and NTSHMDA by random walk for prediction only using the Gaussian interaction profile (GIP) kernel similarity, respectively. Recently, several integrated model methods have also been proposed. For example, Huang et al. (2017) built a computational model by combining two single computational methods (graph-based and neighbor-based models). Qu et al. (2019) constructed an integrated model based on label propagation and matrix decomposition. Peng et al. (2020) developed a reliable negative sample selection method based on the random walk with restart and positive unlabeled learning, then used the logistic matrix factorization with neighborhood regularization for prediction. Yin et al. (2020) also designed an integrated method using label propagation and network consistency projection. Some matrix factorization-based computational methods have been proposed to solve microbe–disease association prediction tasks or similar questions. For example, He et al. (2018) designed a graph regularized non-negative matrix factorization (NMF) framework for prediction. In 2020, Gao et al. (2021) developed multilabel fusion collaborative matrix factorization to solve lncRNA–disease association prediction task. In 2021, Xu et al. (2021a) developed regularized NMF and obtained better prediction results in the lncRNA–protein interaction prediction. However, these models may not achieve better prediction results if the dataset is very sparse.

Some existing methods inevitably have certain limitations. For example, some methods used a single similarity that may cause these methods to be biased toward the fully studied diseases or microbes. Besides, constructions of some algorithms contain many artificial parameters, and it is not easy to select the best parameters for a new dataset, which may reduce the robustness of the model. The imbalance problem of the contribution of microbes and diseases needs to be considered since their numbers are different. The benchmark microbe–disease dataset is very sparse; it is essential to weaken the effect caused by the sparse dataset and let known observed data provide more effective information. Effective methods are still scarce since most MDAs remain unknown (Fan et al., 2019; Long et al., 2021). It is necessary to overcome or weaken these limitations and develop new computational methods to improve prediction performance.

In general, from the algebraic view, biological problems of association prediction could be transformed into matrix completion problems. With the rapid development of machine learning, matrix factorization is a useful tool that has been widely used for matrix completion and solving recommendation system problems. In addition, graph regularization-based methods have been successfully applied to semisupervised learning. Considering some limitations of the previous computation-based methods, to improve the prediction performance, we designed a novel method called CWNMF-GLapRLS for MDA prediction. It used the proposed collaborative weighted NMF technique to recover the sparse association matrix and used the developed graph Laplacian regularized least squares for prediction. The experimental results showed our method achieved superior performance. It is an effective tool to predict potential MDAs and can provide more help for biomedical researchers.

## Materials and Methods

### Dataset

In this study, a widely used benchmark dataset (HMDAD) was used in our experiments. It can be downloaded from http://www.cuilab.cn/hmdad, which was collected by Ma et al. (2017). It contains 292 human microbes, 39 diseases, and 483 experimentally confirmed associations. After filtering out repetitive associations, we obtained 450 associations for prediction. The summary of the microbe–disease association dataset is tabulated in Table 1.

**TABLE 1:** Summary of microbe–disease association dataset.

Types	Statistical information
Microbes	292
Diseases	39
Associations	450
Sparsity (%)	96.05

### Overview of the Proposed Method

**FIGURE 1:**
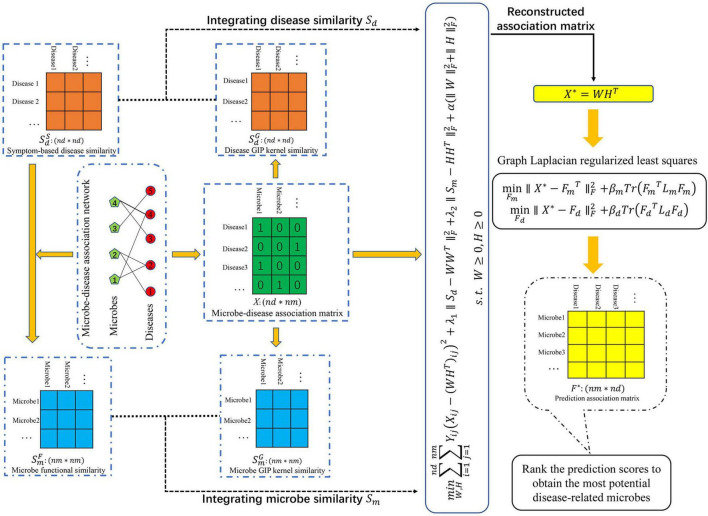
The flowchart of CWNMF-GLapRLS framework for prediction.

To predict potential MDAs, we proposed a novel machine learning-based joint framework named CWNMF-GLapRLS based on the collaborative weighted non-negative matrix factorization (CWNMF) and graph Laplacian regularized least squares (GlapRLS). Figure 1 illustrates the flowchart of the prediction method. It can be decomposed into the following main steps. First, we calculate the functional similarity of microbes through the microbe–disease association network and symptom-based disease similarity. Second, we obtain the GIP kernel similarity based on the topological structure information of the known association matrix, respectively. Third, we calculate the integrated similarities by similarity fusion. Fourth, the proposed CWNMF technique is implemented to reconstruct the association matrix. Finally, we use the designed GlapRLS to score the microbe–disease pairs.

### Similarity Measures

For convenience, we set two sets *D* = {*d*_1_, *d*_2_, …, *d*_*i*_,…*d*_*nd*_} and *M* = {*m*_1_, *m*_2_, …, *m*_*j*_, …, *m*_*nm*_}, which represent all diseases and microbes, where *nd* represents the number of diseases and *nm* denotes the number of microbes. We constructed a binary matrix *XR^nd×nm^* to represent the microbe–disease association network:


(1) $$X\left(i,j\right)=\left\{ \begin{array}{c} 1,ifdiseased_{i}isassociatedwithmicrobem_{j}\\ 0,otherwise \end{array}\right.$$

For disease *d*_*i*_, its interaction profile is represented by *IP*(*d*_*i*_){0, 1}^1*nm^, which denotes the *i*th row of the binary matrix *X*. For microbe *m*_*p*_, its interaction profile is denoted by *IP*(*m*_*p*_){0, 1}^nd*1^, which represents the *p*th column of the binary matrix *X*.

#### Symptom-Based Disease Similarity

Some similarity calculation methods of diseases have been proposed using different kinds of disease information. Symptom-based disease similarity has been increasingly demonstrated that it can provide effective information for MDA prediction (Peng et al., 2018b; Zou et al., 2018). In this work, we also introduced symptom-based disease similarity and utilized $S_{d}^{S}R^{nd\times nd}$ to represent the similarity matrix. $S_{d}^{S}\left(d_{i},d_{j}\right)$ represents the similarity between diseases *d*_*i*_ and *d*_*j*_. More details of the calculation method could be found in a previous study (Zhou et al., 2014). They used a vector of symptoms to represent every disease and used the cosine similarity and term frequency-inverse document frequency (TF-IDF) technique to calculate the similarity of diseases.

#### Microbe Functional Similarity

In this section, inspired by previous work (Zhang et al., 2018; Li et al., 2019) and the basic assumption that microbes will have similar interaction patterns with phenotype diseases that have similar symptoms, we proposed a method to calculate the functional similarity of microbes through the symptom-based disease similarity and association network.

Firstly, we suppose microbes *m*_*i*_ and *m*_*j*_ are associated with *M* and *N* diseases, respectively. Then, set *D*_*i*_ = {*d*_*i*1_, *d*_*i*2_, …, *d*_*ip*_, …, *d*_*iM*_} and *D*_*j*_ = {*d*_*j*1_, *d*_*j*2_, …, *d*_*jq*_, …, *d*_*jN*_} represent two subsets of diseases in the database, in which all diseases are related to the microbe *m*_*i*_ and microbe *m*_*j*_, respectively. Subsequently, we define the microbe functional similarity as follows:


(2) $$\begin{array}{c} \sum_{p=1}^{M}\left(\max_{1\leq q\leq N}S_{d}^{S}\left(d_{ip},d_{jq}\right)\right)+\\ S_{m}^{F}\left(m_{i},m_{j}\right)=\frac{\sum_{q=1}^{N}\left(\max_{1\leq p\leq M}S_{d}^{S}\left(d_{jq},d_{ip}\right)\right)}{M+N} \end{array}$$

where $S_{d}^{S}$ denotes the symptom-based disease similarity matrix; $\max_{1\leq q\leq N}S_{d}^{S}\left(d_{ip},d_{jq}\right)$ represents the maximum similarity score between disease *d*_*ip*_ and all diseases of subset *D*_*j*_; $S_{m}^{F}$ is defined as the microbe functional similarity matrix.

#### Gaussian Interaction Profile Kernel Similarity

In this work, symptom-based disease similarity matrix $S_{d}^{S}$ and microbe functional similarity matrix $S_{m}^{F}$ are both sparse. To integrate more effective information and mine the topology information of known association networks as much as possible, we further introduced popular GIP kernel similarity to calculate the similarity of diseases and microbes (van Laarhoven et al., 2011; Xu et al., 2021b). First, *IP*(*d*_*i*_) of disease *d*_*i*_ and *IP*(*d*_*j*_) of disease *d*_*j*_ were extracted from the training microbe–disease association matrix. Then, we measure the GIP kernel similarity between disease pairs as follows:


(3) $$S_{d}^{G}\left(d_{i},d_{j}\right)=exp\left(-\sigma_{d}{||IP\left(d_{i}\right)-IP\left(d_{j}\right)||}^{2}\right)$$

(4) $$\sigma_{d}=\sigma_{d}^{{}^{\prime }}/\left(\frac{1}{nd}\sum_{k=1}^{nd}{||IP\left(d_{k}\right)||}^{2}\right)$$

where σ_*d*_ is a normalized kernel bandwidth and updated through Eq. (4); $\sigma_{d}^{{}^{\prime }}$ is an adjustment coefficient and was set to *1*; $S_{d}^{G}$ denotes the GIP kernel similarity matrix of diseases.

Similarly, we can calculate the GIP kernel similarity of microbes:


(5) $$S_{m}^{G}\left(m_{p},m_{q}\right)=exp\left(-\sigma_{m}{||IP\left(m_{p}\right)-IP\left(m_{q}\right)||}^{2}\right)$$

(6) $$\sigma_{m}=\sigma_{m}^{{}^{\prime }}/\left(\frac{1}{nm}\sum_{k=1}^{nm}{||IP\left(m_{k}\right)||}^{2}\right)$$

where $\sigma_{m}^{{}^{\prime }}$ is an adjustment coefficient and was set to *1*; σ_*m*_ is a normalized kernel bandwidth and updated through Eq. (6); $S_{m}^{G}$ represents the microbe GIP kernel similarity matrix.

#### Integrated Similarities

Multisimilarity fusion is an effective technique that can fuse different feature information and improve performance. However, the microbe functional similarity matrix is sparse; not every microbe has a functional similarity. It may be unreasonable if the integrated similarity is calculated as a mean of functional similarity and GIP kernel similarity. This approach will dilute the GIP kernel similarity of the integrated similarity. To supplement and integrate more effective biological information for microbes, we defined an integrated similarity for microbes. The calculation of similarity between microbes *m*_*p*_ and *m*_*q*_ is defined as follows:


(7) $$S_{m}\left(m_{p},m_{q}\right)=\left\{ \begin{array}{c} \frac{S_{m}^{F}\left(m_{p},m_{q}\right)+S_{m}^{G}\left(m_{p},m_{q}\right)}{2},ifS_{m}^{F}\left(m_{p},m_{q}\right)\neq 0\\ S_{m}^{G}\left(m_{p},m_{q}\right),otherwise \end{array}\right.$$

where *S*_*m*_*R^nm×nm^* denotes the integrated microbe similarity matrix. Specifically, the final similarity will be calculated as a mean if the microbe pair has a functional similarity. Otherwise, the GIP kernel similarity will be assigned to the integrated similarity.

Similarly, the integrated similarity calculation method of diseases *d*_*i*_ and *d*_*j*_ is defined as follows:


(8) $$S_{d}\left(d_{i},d_{j}\right)=\left\{ \begin{array}{c} \frac{S_{d}^{S}\left(d_{i},d_{j}\right)+S_{d}^{G}\left(d_{i},d_{j}\right)}{2},ifS_{d}^{S}\left(d_{i},d_{j}\right)\neq 0\\ S_{d}^{G}\left(d_{i},d_{j}\right),otherwise \end{array}\right.$$

where *S*_*d*_*R^nd×nd^* denotes the integrated disease similarity matrix.

### Collaborative Weighted Non-negative Matrix Factorization

In general, to recover the association matrix, we could transform this biological problem into a recommendation task. NMF enforced non-negativity constraints on factor matrixes for a low-rank approximation of the non-negative matrix (Lee and Seung, 1999), which could ensure that every element can be represented as an additive linear combination of canonical coordinates. Microbe–disease binary association data *X* is a non-negative matrix. We could use the NMF for matrix completion or association prediction.

In this work, microbe–disease association data *X* is incomplete and sparse. To deal with missing values and effectively overcome the data sparsity problem, we introduced weighted non-negative matrix factorization (WNMF), which slightly changed classical NMF by introducing a weighting term. WNMF was first proposed to cope with missing values in large-scale networks for predicting and representing distances (Mao and Saul, 2004) and has been used for recommendation systems (Gu et al., 2010) to solve the incomplete data problem. The biological problem can be translated into minimizing the following objective:


(9) $$\begin{array}{c} J=\sum_{i=1}^{nd}\sum_{j=1}^{nm}Y_{ij}{\left(X_{ij}-{\left(WH^{\text{T}}\right)}_{ij}\right)}^{2}\\ s.t.W\geq 0,H\geq 0 \end{array}$$

where *XR^nd×nm^* are the training association data; the product of non-negative matrices *WR*^*nd*×*k*^ and *HR*^*nm*×*k*^ is the best approximation of *X*, *k*≪*min*{*nd*, *nm*}. Microbes and diseases are mapped into a shared latent space with a low-dimensionality *k*. *Y* is a non-negative weight matrix used to reduce the influence of missing values on matrix factorization, where *Y*_*ij*_ = 0 indicates *X*_*ij*_ is a missing value and *Y*_*ij*_ = 1 indicates *X*_*ij*_ is an observed value. The objective function will degenerate into the standard NMF when all weights of matrix *Y* are equal to one.

In 2000, Lee and Seung (2001) have shown that the iterative update algorithm can ensure NMF objective function convergence and is very easy to use and code. At the same time, an iterative multiplicative updating algorithm was also used to solve WNMF (Zhang et al., 2006). The objective function leads to the following updated formulas:


(10) $$w_{ik}=w_{ik}\frac{{\left(Y⊙XH\right)}_{ik}}{{\left(Y⊙\left(WH^{\text{T}}\right)H\right)}_{ik}}$$

(11) $$h_{jk}=h_{jk}\frac{{\left({\left(Y⊙X\right)}^{\text{T}}W\right)}_{jk}}{{\left({\left(Y⊙\left(WH^{\text{T}}\right)\right)}^{\text{T}}W\right)}_{jk}}$$

where ⊙ is the Hadamard product. These updated rules are computationally efficient.

In 2021, Xu et al. (2021a) developed regularized NMF and obtained better prediction results in the lncRNA–protein interaction prediction. This study proved that collaborative factorization of the similarity matrix can effectively guide matrix factorization and improve prediction performance. To introduce more effective similarity information to guide the matrix factorization, two collaborative regularization terms were incorporated into the WNMF framework to fuse similarity information and constrain two low-dimensional representations. It can be turned into a constrained optimization problem and formulated a joint matrix factorization framework of association data and similarity data. Then, we can obtain a novel objective function as follows:


(12) $$\begin{array}{c} J=\sum_{i=1}^{nd}\sum_{j=1}^{nm}Y_{ij}{\left(X_{ij}-{\left(WH^{\text{T}}\right)}_{ij}\right)}^{2}\\ \;+\lambda_{1}{∥S_{d}-WW^{\text{T}}∥}_{F}^{2}+\lambda_{2}{∥S_{m}-HH^{\text{T}}∥}_{F}^{2}\\ \;s.t.W\geq 0,H\geq 0 \end{array}$$

where ||⋅||_*F*_ is the Frobenius norm; λ_1_ and λ_2_ are non-negative regularization parameters balancing two collaborative regularization terms and the reconstruction error. The objective function will degenerate into WNMF if λ_1_ and λ_2_ are equal to zero.

To prevent overfitting and adjust the smoothness of *W* and *H*, we introduced the Tikhonov (*L*_2_) regularization terms (Xiao et al., 2018) into the objective function and obtained the final collaborative weighted non-negative matrix factorization (CWNMF) objective function as follows:


(13) $$\begin{array}{c} J=\sum_{i=1}^{nd}\sum_{j=1}^{nm}Y_{ij}{\left(X_{ij}-{\left(WH^{\text{T}}\right)}_{ij}\right)}^{2}\\ \;+\lambda_{1}{∥S_{d}-WW^{\text{T}}∥}_{F}^{2}+\lambda_{2}{∥S_{m}-HH^{\text{T}}∥}_{F}^{2}\\ \;+\alpha \left({∥W∥}_{F}^{2}+{∥H∥}_{F}^{2}\right)\\ \;s.t.W\geq 0,H\geq 0 \end{array}$$

where α is used to adjust the Tikhonov regularization terms, which is a regularization coefficient. To improve the robustness of the model, we set the same value for the same Tikhonov regularization terms, and α was set to 1 for the dataset.

Since the objective function is not convex in both variables *W* and *H*, the iterative update algorithm was used to search the local minimum. Here, we used the Lagrange multipliers method and Karush–Kuhn–Tucker (KKT) conditions to optimize the objective function. Eventually, we obtained the following multiplicative updates:


(14) $$w_{ik}=w_{ik}\frac{{\left(Y⊙XH+2\lambda_{1}S_{d}W\right)}_{ik}}{{\left(Y⊙\left(WH^{\text{T}}\right)H+\alpha W+2\lambda_{1}WW^{\text{T}}W\right)}_{ik}}$$

(15) $$h_{jk}=h_{jk}\frac{{\left({\left(Y⊙X\right)}^{\text{T}}W+2\lambda_{2}S_{m}H\right)}_{jk}}{{\left({\left(Y⊙\left(WH^{\text{T}}\right)\right)}^{\text{T}}W+\alpha H+2\lambda_{2}HH^{\text{T}}H\right)}_{jk}}$$

Then, we can obtain the reconstructed association matrix X* = *WH*^T^. The low-dimensionality representation *k* was set as 35 in the process of prediction.

### Graph Laplacian Regularized Least Squares

In this section, to improve the prediction performance, we developed a semisupervised learning method named graph Laplacian regularized least squares based on the reconstructed association matrix X*. Graph regularization is used to fully exploit data geometric structure for semisupervised learning. Specifically, in the prediction space of microbes, with the above defined integrated microbe similarity matrix *S*_*m*_, the graph Laplacian regularization term was incorporated into the least-squares framework to enhance the learning performance. The optimization problem can be formularized as follows:


(16) $$\min_{F_{m}}{∥X^{*}-F_{m}^{\text{T}}∥}_{F}^{2}+\beta_{m}\frac{1}{2}\left(\sum_{i,j=1}^{nm}{∥F_{mi}-F_{mj}∥}^{2}S_{m_{ij}}\right)$$

where X*R^nd×nm^ is a reconstructed association matrix obtained by the CWNMF method; β_*m*_ is the regularization coefficient; *F*_*m*_ is the prediction score matrix based on the microbes; *F*_*mi*_ denotes the *i*th row of *F*_*m*_ ∈ *R^nm×nd^*; and *F*_*mj*_ denotes the *j*th row of *F*_*m*_. The graph Laplacian regularization term (Xiao et al., 2018; Cai et al., 2020) can be transformed into a matrix form by some algebraic manipulations:


(17) $$\frac{1}{2}\left(\sum_{i,j=1}^{nm}{∥F_{mi}-F_{mj}∥}^{2}S_{m_{ij}}\right)=Tr\left(F_{m}^{\text{T}}L_{m}F_{m}\right)$$

where *Tr*(?) denotes the trace of a matrix; *L*_*m*_ = *D*_*m*_−*S*_*m*_ is the graph Laplacian matrix for *S*_*m*_. *D*_*m*_ is the diagonal matrix whose entries are calculated as the column sums of *S*_*m*_. Therefore, Eq. (16) can be transformed into the following equation:


(18) $$\underset{F_{m}}{\text{min}}{∥X^{*}-F_{m}^{\text{T}}∥}_{F}^{2}+\beta_{m}Tr\left(F_{m}^{\text{T}}L_{m}F_{m}\right)$$

where *F*_*m*_ = *S*_*m*_α_*m*_, α_*m*_ ∈ *R^nm×nd^* is a matrix (Xia et al., 2010). To improve the robustness of the model and according to the choice of previous similar work (van Laarhoven et al., 2011), β_*m*_ was set to 1. We can obtain the solution of the optimization problem by some manipulations, $\alpha_{m}^{*}={\left(S_{m}+L_{m}S_{m}\right)}^{-1}X^{*\text{T}}$. Then, in the microbe prediction space, the prediction score matrix can be calculated as follows:


(19) $$F_{m}=S_{m}{\left(S_{m}+L_{m}S_{m}\right)}^{-1}X^{*\text{T}}$$

Similarly, for disease prediction space, the optimization problem can be formularized as the following equation:


(20) $$\underset{F_{d}}{\text{min}}{∥X^{*}-F_{d}∥}_{F}^{2}+\beta_{d}Tr\left(F_{d}^{\text{T}}L_{d}F_{d}\right)$$

where β_*d*_ was also set to 1. We can obtain the prediction score matrix in the disease prediction space.


(21) $$F_{d}=S_{d}{\left(S_{d}+L_{d}S_{d}\right)}^{-1}X^{*}$$

Finally, the predicted microbe–disease association matrix is calculated as $F^{*}=\eta F_{m}^{\text{T}}+\left(1-\eta \right)F_{d}$, where η is a tradeoff parameter describing the importance of microbe and disease space. The microbe-related diseases can be prioritized by the size of the prediction scores in matrix F*. The detailed steps of the CWNMF-GlapRLS procedure are detailed in Algorithm 1.

**Algorithm 1. CWNMF-GlapRLS Algorithm.**

**Input:** Matrices *XR^nd×nm^*, *S*_*d*_*R^nd×nd^* and *S*_*m*_*R^nm×nm^*; non-negative weight matrix *YR^nd×nm^*; regularization coefficients λ_1_ and λ_2_; tradeoff parameter η.

**Output:** Predicted score matrix F*.

Randomly initialize two non-negative matrices *WR^nd×k^* and *HR^nm×k^*.

Repeat

Update *W* and *H* by the following rules:

$w_{ik}=w_{ik}\frac{{\left(Y⊙XH+2\lambda_{1}S_{d}W\right)}_{ik}}{{\left(Y⊙\left(WH^{\text{T}}\right)H+\alpha W+2\lambda_{1}WW^{\text{T}}W\right)}_{ik}}$

$h_{jk}=h_{jk}\frac{{\left({\left(Y⊙X\right)}^{\text{T}}W+2\lambda_{2}S_{m}H\right)}_{jk}}{{\left({\left(Y⊙\left(WH^{\text{T}}\right)\right)}^{\text{T}}W+\alpha H+2\lambda_{2}HH^{\text{T}}H\right)}_{jk}}$

Until convergence

Reconstruct association matrix X* = *WH*^T^.

Calculate diagonal matrix *D*_*m*_;

*L*_*m*_ = *D*_*m*_−*S*_*m*_;

*F*_*m*_ = *S*_*m*_(*S*_*m*_ + *L*_*m*_*S*_*m*_)^−1^*X*^T^//calculate the score matrix *F*_*m*_ based on the microbe prediction space.

Calculate diagonal matrix *D*_*d*_; *L*_*d*_ = *D*_*d*_−*S*_*d*_;

*F*_*d*_ = *S*_*d*_(*S*_*d*_ + *L*_*d*_*S*_*d*_)^−1^*X*//calculate the score matrix *F*_*d*_ based on the disease prediction space.

Return $F^{*}=\eta F_{m}^{\text{T}}+\left(1-\eta \right)F_{d}$.

## Results

### Evaluation Metrics

To ensure the reliability of experimental results, we implemented the global leave-one-out cross-validation (LOOCV) framework to validate the performance of models (Bao et al., 2017). In each round cross-validation of the LOOCV framework, the integrated similarity of diseases and microbes should be recalculated, which can guarantee independence between the validation set and the training set. Specifically, under this framework, every known microbe–disease pair will be regarded as a test set, the rest of the known pairs are treated as the training set in the dataset, and all pairs without observed association are used as candidate samples. We calculated the predicted microbe–disease score matrix by running the model. Then, the prediction score is compared with all candidate samples to get the ranking of each test sample. This testing sample will be regarded as a successful prediction if the rank is higher than the threshold. We used the receiver operating characteristic (ROC) curve to vividly describe the performance of the model by calculating sensitivity (true positive rates) and 1-specificity (false positive rates) with different thresholds. In addition, we calculated the area under curve (AUC) to intuitively describe the performance. Similarly, fivefold cross-validation (CV) was also applied to evaluate the effectiveness of the models. The experiment was repeatedly performed 10 times to reduce potential bias caused by random segmentation of the dataset. At the same time, the ROC curves and average AUC values were also obtained under the fivefold CV framework.

### Parameter Sensitivity and Model Setting

**FIGURE 2:**
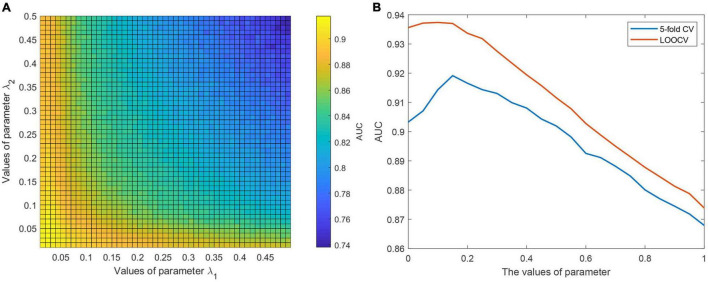
**(A)** The illustration of determining the optimal values of parameter pair (λ_1_, λ_2_) under grid search. **(B)** The effects between parameter η and AUC value.

It is necessary to evaluate the influence of model parameters on the prediction performance of CWNMF-GLapRLS. We studied the influence of two regularization parameters λ_1_ and λ_2_. The grid search method was adopted to find better model parameters. In the experiments, we first tuned the range of two parameters from 0 to 0.5, and each step is 0.01. Then, the proposed method was run to find the optimal model parameter values based on the AUC values on the 50×50 grid. Figure 2A shows the relationship between the AUC value and the parameter pair (λ_1_, λ_2_) under the fivefold framework. Finally, we selected the parameter pair of (0.02, 0.04) as the optimal value of (λ_1_, λ_2_) based on the grid search results under the two evaluation frameworks. Then, we fixed the parameter pair and adjusted the parameter η. The effects between parameter η and the AUC value are shown in Figure 2B. Finally, η was set at 0.15 as the optimal value for the following analysis.

**FIGURE 3:**
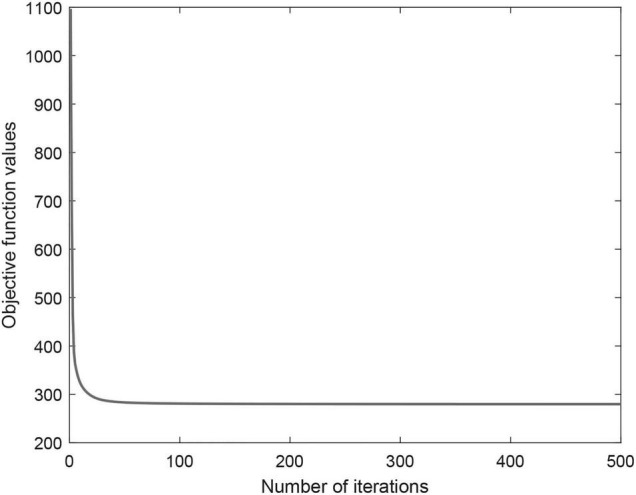
Convergence behavior of CWNMF objection function.

Iterative update algorithm can ensure objective function convergence and guarantee to converge to a locally optimal. Figure 3 shows the objective function convergence curve of CWNMF. From the figure, we can see that the convergence is fast, and the objective function value decreases as the iterations. The number of iterations is usually very small (fewer than 100) before practical convergence. Thus, the proposed method can scale to larger datasets. Finally, the number of iterations was set at 300 in the process of prediction.

### Performance Analysis

**FIGURE 4:**
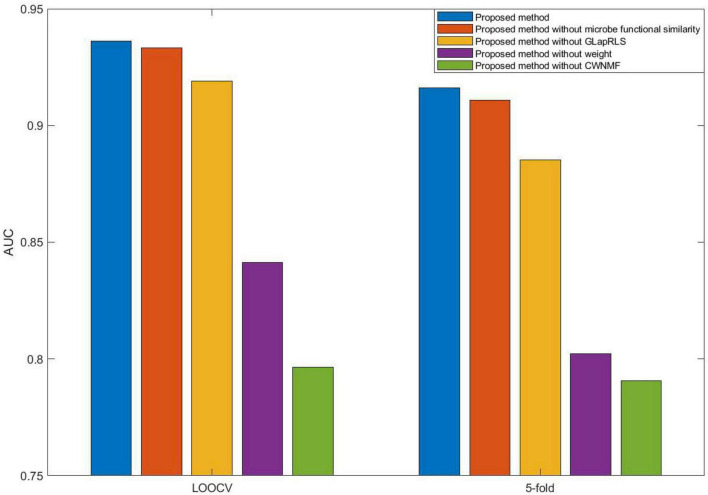
The performance comparison of different methods.

Here, we compared five different forms (proposed method, proposed without microbe functional similarity, proposed method without weight, proposed method without GLapRLS, and proposed method without CWNMF) of the introduced method to analyze the proposed method. Especially, to improve the prediction performance and fuse more similarity information, we calculated microbe functional similarity. To deal with missing values and effectively overcome the data sparsity problem, we introduced WNMF, which slightly changed classical NMF by introducing a weighting term, and proposed the technique CWNMF for recovering the association matrix. The proposed method is a joint framework. The CWNMF technique was first used to recover the original matrix; then, the GLapRLS method was used for prediction. Figure 4 shows the performance comparison of methods with different forms on the HMDAD dataset. The proposed method performs better than the other four methods. From the figure, we can obtain that the combination of CWNMF and GLapRLS can significantly improve the prediction performance. The comparison results indicate that microbe functional similarity and weighting term are also effective in improving the performance of prediction.

### Comparison With State-of-the-Art Prediction Methods

In this section, to evaluate the effectiveness of the proposed method, we compared it with 5 state-of-the-art methods, including graph regularized non-negative matrix factorization (GRNMFHMDA) (He et al., 2018), KATZ measure (KATZHMDA) (Chen et al., 2017), bi-random walk (BiRWHMDA) (Zou et al., 2017), Laplacian regularized least squares (LRLSHMDA) (Wang et al., 2017), and network topological similarity (NTSHMDA) (Luo and Long, 2020) for human MDA prediction methods. Optimal parameter combinations for 5 comparison methods are listed in Supplementary Table 1.

**FIGURE 5:**
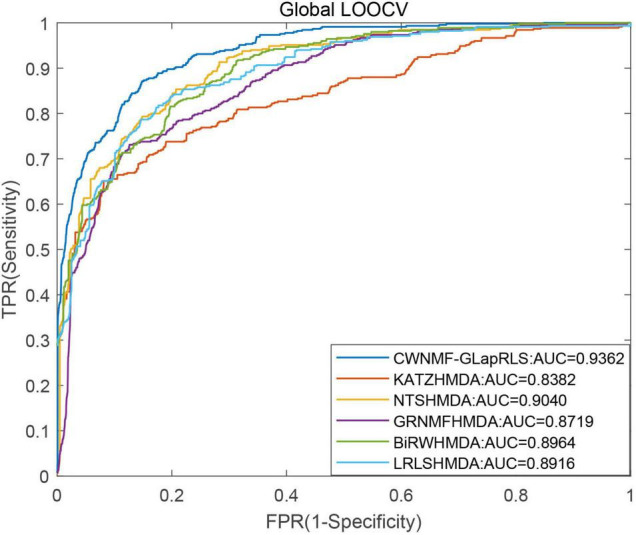
The ROC curves and AUC values of six methods under LOOCV framework.

**FIGURE 6:**
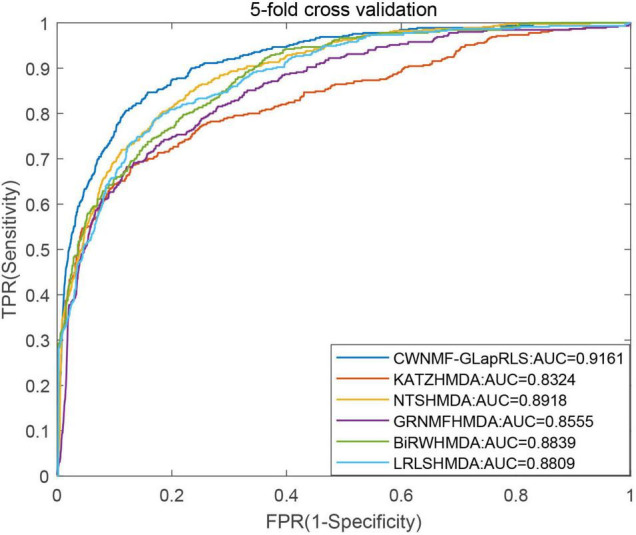
The ROC curves and average AUC values of six methods under fivefold CV framework.

First, under the LOOCV framework, the ROC curves and AUC values of six methods have been shown in Figure 5. From the figure, we can see that the proposed method outperforms other methods with an AUC of 0.9362 under the LOOCV framework, while GRNMFHMDA, KATZHMDA, LRLSHMDA, BiRWHMDA, and NTSHMDA obtained AUC values of 0.8719, 0.8382, 0.8916, 0.8964, and 0.9040, respectively. In addition, the ROC curves and average AUC values of six methods under the fivefold CV framework have been shown in Figure 6. We can see that the proposed method is more outstanding than other methods with an AUC of 0.9161 under the fivefold CV framework, while GRNMFHMDA, KATZHMDA, LRLSHMDA, BiRWHMDA, and NTSHMDA obtained AUC values of 0.8555, 0.8324, 0.8809, 0.8839, and 0.8918, respectively. These experimental results proved that our method is effective and reliable, and may be an effective tool for seeking potential disease-related microbes.

### Case Studies

Accumulating evidence has shown that the development and occurrence of human disease are closely related to the imbalance of the microbial community. To infer potential association, in this section, case studies were implemented on two different common human diseases (asthma and IBD). In this way, we used the number of validated predicted microbes of the top 15 prediction results to further measure the predictive capability, respectively. If the genus of a microbe is related to the disease, this microbe will be related to the disease. This assumption has been widely used in related studies (Niu et al., 2019; Wang et al., 2019). Specifically, for a given disease, all pairs without observed association were regarded as candidate samples. We calculated the association scores for all microbes based on the joint framework. All candidate microbe samples were prioritized based on their scores.

Asthma is a common chronic inflammatory disease, which affects the daily lives of 300 million people worldwide (Lambrecht and Hammad, 2015). To investigate asthma-causing microbes, the prediction results have been tabulated in Table 2. There are 13 out of the top 15 candidate microbes that have been successfully supported to be associated with asthma based on previously published medical or biological literature. According to the table, our method has an excellent effect. Increasing evidence has shown that the development and occurrence of human asthma are closely related to the imbalance of the microbial community. For example, some clinical evidence has shown that asthmatic patients have lower Actinobacteria, Firmicutes, and Bacteroides proportions (Björkstén et al., 1999; Marri et al., 2013). The colonization by *Clostridium coccoides* subcluster XIVa species at age 3 weeks may serve as an early indicator of possible asthma (Vael et al., 2011). In addition, colonization by *Clostridium difficile* at age 1 month was closely associated with asthma at 6–7 years old (Van Nimwegen et al., 2011). One study showed that *Streptococcus* increases the risk of asthma by early asymptomatic colonization (Teo et al., 2015). *Lactobacillus* has been shown to be beneficial to asthmatic children (Huang et al., 2018).

**TABLE 2:** Prediction results of the top 15 asthma-associated microbes.

Rank	Microbe	Evidence
1	*Firmicutes*	PMID:23265859
2	*Clostridium coccoides*	PMID:21477358
3	*Actinobacteria*	PMID:26220531
4	*Clostridia*	Unconfirmed
5	*Bacteroides*	PMID:10202341
6	*Clostridium difficile*	PMID:21872915
7	*Lactobacillus*	PMID:30400588
8	*Bifidobacterium*	PMID:24735374
9	*Lachnospiraceae*	PMID:31958431
10	*Veillonella*	PMID:26424567
11	*Streptococcus*	PMID:25865368
12	*Staphylococcus aureus*	PMID:25533526
13	*Fusobacterium nucleatum*	Unconfirmed
14	*Faecalibacterium prausnitzii*	PMID:30208875
15	*Fusobacterium*	PMID:27838347

IBD starts with inflammation and is a collective term for a wide range of intestinal diseases, which is a worldwide healthcare problem (Hossen et al., 2020). IBD has become one of the most studied human diseases linked to gut microbiota (Kostic et al., 2014). We listed the top 15 IBD-associated microbes in Table 3. As a result, 14 out of the top 15 candidate microbes have been successfully validated to be associated with the IBD based on published literature. Emerging evidence showed that many microbes are closely related to IBD. For example, the infection of *Clostridium difficile* is a significant clinical challenge for IBD patients, which can result in morbidity and mortality (Hashash and Binion, 2014). Some studies showed *Bacteroidetes, Bacteroides, Firmicutes*, and *Prevotella* are associated with the development of IBD (Juste et al., 2014; Walters et al., 2014). In IBD patients, *Prevotella*, *Veillonella*, and *Haemophilus* were found, which can contribute largely to dysbiosis, which is associated with inflammatory responses (Said et al., 2014). The study confirmed that *Helicobacter pylori* was inversely associated with IBD (Sonnenberg and Genta, 2012). In addition, *Veillonella* and *Bifidobacterium* decreased, while the proportion of *Lactobacillus* increased in the feces of IBD patients (Takaishi et al., 2008). Case studies indicated that our method has a practical effect on potential association prediction.

**TABLE 3:** Prediction results of the top 15 IBD-associated microbes.

Rank	Microbe	Evidence
1	*Bacteroidetes*	PMID:25307765
2	*Prevotella*	PMID:25307765
3	*Firmicutes*	PMID:25307765
4	*Clostridium coccoides*	PMID:19235886
5	*Helicobacter pylori*	PMID:22221289
6	*Bacteroides*	PMID:25307765
7	*Clostridia*	PMID:31142855
8	*Haemophilus*	PMID:24013298
9	*Clostridium difficile*	PMID:24838421
10	*Lactobacillus*	PMID:24478468
11	*Bifidobacterium*	PMID:24478468
12	*Veillonella*	PMID:24013298
13	*Staphylococcus aureus*	PMID:19809406
14	*Staphylococcus*	Unconfirmed
15	*Faecalibacterium prausnitzii*	PMID:32815163

## Conclusion and Discussion

Studies investigating microbiomes demonstrated a critical role for microbes in human health and disease. Identifying potential disease-related microbes is essential for understanding the mechanisms of host–microbe interactions and revealing the pathological mechanism of human diseases. Here, we designed a joint framework for association prediction based on the proposed CWNMF and graph Laplacian regularized least squares. The experimental results showed that our method achieved the best performance by comparing it with 5 state-of-the-art models. Case studies of asthma and IBD also further demonstrated that the proposed method is a useful tool to infer potential associations. All experimental results adequately demonstrated that the proposed method has reliable and effective prediction performance.

There are several key factors that make the proposed method have effective performance. Firstly, compared with graph regularized NMF and collaborative matrix factorization, we introduced a weighting term and changed the NMF for prediction to deal with missing values and weaken the effect caused by a sparse dataset. Secondly, we calculated the functional similarity of microbes and introduced symptom-based disease similarity for fusing more similarity information. Thirdly, to restructure the sparse association matrix, two collaborative regularization terms were incorporated into the framework to fuse similarity information and constrain two low-dimensional representations, guiding the matrix factorization process. We used the iterative update algorithm to solve the matrix factorization objective function, which is easy to use and code. Semisupervised learning provides more effective information in the process of prediction. We hope that the proposed method can help biomedical researchers conduct follow-up research, and a growing number of potential disease-related microbes could be verified through biological or clinical experiments.

## Data Availability Statement

The original contributions presented in the study are included in the article/Supplementary Material, further inquiries can be directed to the corresponding author/s.

## Author Contributions

DX: methodology, software, formal analysis, and writing—original draft, writing—review and editing. HX: data curation, methodology, software, and writing—original draft. YZ: supervision, funding acquisition, funding acquisition, and writing—review and editing. RG: formal analysis, supervision, and funding acquisition. All authors contributed to the article and approved the submitted version.

## Conflict of Interest

The authors declare that the research was conducted in the absence of any commercial or financial relationships that could be construed as a potential conflict of interest.

## Publisher’s Note

All claims expressed in this article are solely those of the authors and do not necessarily represent those of their affiliated organizations, or those of the publisher, the editors and the reviewers. Any product that may be evaluated in this article, or claim that may be made by its manufacturer, is not guaranteed or endorsed by the publisher.
