# Supplementary material for: Novel Collaborative Weighted Non-negative Matrix Factorization Improves Prediction of Disease-Associated Human Microbes
Source: Front Microbiol. 2022 Mar 10;13:834982. doi: 10.3389/fmicb.2022.834982 (PMC8965656; doi:10.3389/fmicb.2022.834982)
Supplement: Supplementary file 1 [file Table_1.DOCX]

**Supplementary material**

In this paper, to evaluate the effectiveness of the proposed method, we compared it with 5 state-of-the-art methods, including Graph Regularized Nonnegative Matrix Factorization (GRNMFHMDA) (He et al., 2018), KATZ measure (KATZHMDA) (Chen et al., 2017), Bi-Random Walk (BiRWHMDA) (Zou et al., 2017), Laplacian Regularized Least Squares (LRLSHMDA) (Wang et al., 2017) and Network Topological Similarity (NTSHMDA) (Luo and Long, 2020) for human MDA prediction methods.

In this appendix, we provided the parameters for the 5 state-of-the-art prediction methods.

**Table S1** Optimal parameter combinations for 5 comparison methods

| Methods | Parameters |
| --- | --- |
| KATZHMDA | $\gamma^{'}=1$, $\beta=0.01$,$k=2$ |
| NTSHMDA | $\gamma=0.7$, $\varphi=0.9,$ $\delta=0.3$ |
| GRNMFHMDA | $\lambda_{l}=1$, $\lambda_{m}=1$, $\lambda_{d}=1$ |
| BiRWHMDA | $\alpha=0.4$, $l=2$, $\gamma=2$ |
| LRLSHMDA | $\eta M=1$, $\eta D=1$, $lw=0.4$ |

# References

He, B. S., Peng, L. H., and Li, Z. (2018). Human microbe-disease association prediction with graph regularized non-negative matrix factorization. Front. Microbiol. 9, 1–11. doi:10.3389/fmicb.2018.02560.

Chen, X., Huang, Y. A., You, Z. H., Yan, G. Y., and Wang, X. S. (2017). A novel approach based on KATZ measure to predict associations of human microbiota with non-infectious diseases. Bioinformatics 33, 733–739. doi:10.1093/bioinformatics/btw715.

Zou, S., Zhang, J., and Zhang, Z. (2017). A novel approach for predicting microbe-disease associations by bi-random walk on the heterogeneous network. PLoS One 12, 1–16. doi:10.1371/journal.pone.0184394.

Wang, F., Huang, Z. A., Chen, X., Zhu, Z., Wen, Z., Zhao, J., et al. (2017). LRLSHMDA: Laplacian regularized least squares for human microbe-disease association prediction. Sci. Rep. 7, 1–11. doi:10.1038/s41598-017-08127-2.

Luo, J., and Long, Y. (2020). NTSHMDA: Prediction of Human Microbe-Disease Association Based on Random Walk by Integrating Network Topological Similarity. IEEE/ACM Trans. Comput. Biol. Bioinforma. 17, 1341–1351. doi:10.1109/TCBB.2018.2883041.
